# 

**Published:** 1873-07

**Authors:** 


### Books and Periodicals.

"FUNCTION AND DISEASES OF WOMEN," is the title of a neatly printed and bound volume written and published by L. C. Warner, A. M., M. D., of Cortland, N. Y. Dr. Warner has had an extensive experience in the management of diseases peculiar to women, which has enabled him to write a very valuable and attractive work upon the subject. We welcome it as one of the few popular books upon health matters that it is safe to recommend to the public. The price is $2, to be had of the author, address above given.

---

THE PHILADELPHIA MEDICAL TIMES, published by J. B. Lippincott & Co., 716 Market St., Phila., at five dollars a year, is the best investment that any medical man can make at the price. We regard it as the best medical periodical in the country.

---

PETERSON'S LADIES' MAGAZINE, fresh and sprightly, reaches our table monthly, and is eagerly watched for by our lady patients.—They tell us it is a very cheap and remarkably interesting journal. Price $2 a year.

---

THE ALDINE.—Have you seen it? Are you fond of beautiful engravings, elegant typography and refined literature? If so, by all means subscribe for the Aldine. It is a large, quarto magazine, published monthly by Jas. Sutton & Co., No. 58 Maiden Lane, N. Y., at five dollars a year, with two large oil chromos free. To be had only of the publishers.

---

LOCKE'S NATIONAL MONTHLY.—A magazine of American and Foreign Literature, and edited by D. R. Locke, the renowned *Nasby*. It is only a dollar a year, and is a first class magazine in every particular.

---

NÆVUS.—A pamphlet upon this subject is published by J. H. Pooley, M. D., of Yonkers, N. Y., and is a valuable contribution to surgical science.

---

THE VIRGINIA CLINICAL RECORD, a monthly medical journal, edited by J. S. Dorsey Cullen, M. D., at Richmond Va., is one of the very best of southern medical journals. M. W. Hazlewood, publisher, $2 a year.

### SUBSCRIPTIONS RECEIVED.

In this column we will credit every subscription received during the last quarter. Subscribers will please notify us immediately if their payments are not duly credited. The State only is given—this, with the full name of the party subscribing, will be sufficient to indicate who is meant:

NEW YORK.—S. M. Fassett, E. St. John, A. F. Adams, James Chapman, Mrs. L. Lowman, C. L. Fitch, A. B. Chamberlain, Hon. S. H. Ainsworth, Chas. Mudge, Dr. S. O. Gleason, E. Harden, C. L. Harrington, Mrs. M. G. Ennis, J. B. Frasier, E. R. Brundage, Dr. C. S. Vandervere, DeWitt Baker, Dr. J. A. Shannon, J. W. Henocksburg, C. Rathbone, Dr. Crandall.

PENNSYLVANIA.—Mrs. J. W. Chapman, T. S. Norton, J. C. Schaffer, S. G. Fry, Dr. D. McDonnell, P. Hillbish, B. F. Antrim, Dr. R. Moody, Dr. J. P. Marsh, Dr. L. M. Snyder, Dr. H. P. Smith, Dr. L. Gearhart, Dr. P. Arlington, Dr. J. H. Shearer, Mrs. J. Farenbaugh.

ALABAMA.—Dr. S. S. Thrower, H. D. Curtis.

ARKANSAS.—Dr. J. H. Carroll, Drs. Hooper and Braysacher, J. H. Beveridge, U. M. Morey.

CALIFORNIA.—Dr. J. F. Gibbon.

DISTRICT COLUMBIA.—A. Zooler.

INDIANA.—Dr. J. S. Athon, Dr. J. H. Moore.

MICHIGAN.—G. R. Kinsman, Mrs. A. Ransome, Mrs. S. Paine.

NEW JERSEY.—J. M. Atwater.

OHIO.—Dr. B. S. Leonard.

OREGON.—Dr. T. N. Snow.

SOUTH CAROLINA.—B. Grimball.

TENNESSEE.—Dr. A. A. Davidson.

TEXAS.—Dr. A. R. Kirkpatrick, Jas. B. Long, Dr. F. L. Dickenson, E. Heppenstall.

THE BISTOURY,

ELMIRA, N. Y.

### A USEFUL PREMIUM.

For one hundred and ten subscribers to THE BISTOURY, at *Fifty Cents each*, amounting to $55,00, we will give a

### Grover & Baker Sewing Machine,

***Costing $55,00!***

Everybody knows what a GROVER & BAKER Machine is, so that it requires no puffing. We will guarantee them to be new, and in perfect and complete order.

For twenty subscribers, at fifty cents each, we will send to the getter up of the club, one of

### McAllister's New Microscopes,

The price of which is Five Dollars.

For seventy subscribers we will give as a premium a good, silver, hunting case, AMERICAN WATCH, worth $35,00.

Those who prefer to work for money, can do so according to our

### CASH LIST.

Examine it and learn *How to make from ten to twenty dollars a day!*

| | | | | | | |
|---|---|---|---|---|---|---|
| For | 100 | Subscribers and | $50,00 | you may | retain | $25,00 |
| " | 50 | " | 25,00 | " | " | 10,00 |
| " | 25 | " | 12,50 | " | " | 5,00 |
| " | 10 | " | 5,00 | " | " | 2,00 |
| " | 5 | " | 2,50 | " | " | 1,00 |

So that you get your premiums in SOLID CASH, instead of cheap books, worthless pictures, or "pinched beck" jewelry.

### THE WAY TO REMIT TO US.

Collect $50 from 100 Subscribers. Then put $25 of it in your own pocket for your work and buy a Post Office order or draft with the other $25, and remit it to us. Also, send the names and address, (Post Office, County and State,) of each subscriber, PLAINLY WRITTEN, when they will receive THE BISTOURY by return mail, which will be an evidence to them that your contract has been faithfully performed. For fifty subscribers you will send us $15, you retaining $10, and so on, down through the list.

Women, who are worn out with sewing, throw down your needles, take a copy of THE BISTOURY and solicit subscriptions from your neighbors. You will find it a healthier and more profitable employment.

Post Masters can, without trouble, secure large lists at their offices, and make it pay better than their salaries.

Young men, out of employment, can earn a suit of clothes in one day, and make their canvassing pay them ever afterwards.

Boys and girls can canvass for THE BISTOURY,

**AND MAKE MORE MONEY**

than they ever dreamed of possessing before.

Try it, everybody. There is money in it, and

**You have your Pay in your own hands.**

Send all sums over $5 by Post Office Order or Draft, made payable to THAD S. UP DE GRAFF, M. D. Smaller sums may be sent in currency.

☞ Those who desire to get up Clubs, will have sample copies sent them FREE, by writing for them. ADDRESS,

**THE BISTOURY,**
**ELMIRA, N. Y.**

### AN ELEGANT PREMIUM!

The Young Folks' Rural, Two Chromos & The Bistoury,

ALL FOR

**ONE DOLLAR AND FIFTY CENTS.**

THE YOUNG FOLKS' RURAL is a rural and literary monthly journal, for young people of country and city. It is a beautifully printed, and elegantly illustrated sixteen page paper, brim full of entertaining stories, sketches, puzzles, plays, enigmas, charades, riddles, etc., etc., of surpassing interest to young people. We regard it as the very best youth's journal published in this country. It is published in Chicago, by H. N. F. Lewis, at $1.50 a year. Two elegant Chromos given to every subscriber.

We offer it. together with Chromos and Bistoury, one year, for $1,50.

Address, **THE BISTOURY,**
ELMIRA, N. Y.

### THE BISTOURY GIVEN AWAY.

OUR TERMS FOR CLUBBING WITH OTHER JOURNALS.

We will cause any of the publications below to be sent together with THE BISTOURY, for one year, on the following terms:—

| | PUB. PRICE. | WITH BISTOURY. |
|---|---|---|
| American Agriculturalist | $1 50 | $1 50 |
| Scribner's Monthly | 4 00 | 4 00 |
| Peterson's Ladies' Magazine | 2 00 | 2 00 |
| Home and Health | 1 50 | 1 50 |
| Little Corporal | 1 50 | 1 50 |
| Good Health | 2 00 | 2 00 |
| Atlantic Monthly | 4 00 | 4 00 |
| Western Rural | 2 00 | 2 00 |
| Young Folks' Rural | 1 50 | 1 50 |
| Peter's Musical Monthly | 3 00 | 3 00 |
| Eclectic Magazine | 5 00 | 5 00 |
| The Galaxy | 4 00 | 4 00 |
| Hearth and Home | 4 00 | 4 00 |
| Godey's Lady's Book | 3 00 | 3 00 |
| Wood's Household Magazine | 1 00 | 1 00 |
| The Children's Hour | 1 50 | 1 50 |
| Saturday Evening Post | 2 50 | 2 50 |
| Phrenological Journal | 3 00 | 3 00 |
| Medical Times, Philadelphia (Weekly) | 5 00 | 5 00 |
| The London Medical Record (Weekly) | 5 00 | 5 00 |
| Merry's Musem | 1 50 | 1 50 |
| Ballou's Monthly | 1 50 | 1 50 |
| Arthur's Home Magazine | 2 50 | 2 50 |
| Monthly Novelet | 2 00 | 2 00 |
| Our Young Folks | 2 00 | 2 00 |
| Ohio Farmer | 2 00 | 2 00 |
| Cultivator and Country Gentleman | 2 50 | 2 50 |
| New York Independent | 2 50 | 2 50 |
| American Union | 2 50 | 2 50 |
| Rural New Yorker | 3 00 | 3 00 |
| Saturday Night | 3 00 | 3 00 |
| Ladies' Repository | 3 50 | 3 50 |
| Harper's Magazine | 4 00 | 4 00 |
| " Bazar | 4 00 | 4 00 |
| " Weekly | 4 00 | 4 00 |
| Every Saturday | 5 00 | 5 00 |
| North American Review | 6 00 | 6 00 |
| Lippincott's Magazine | 4 00 | 4 00 |
| American Farmer's Advocate | 1 00 | 1 00 |
| Toledo Weekly Blade | 2 00 | 2 00 |

Forward the price of any of the above publications to our address, and you will receive both journals regularly, for one year. Address

**The Bistoury,** ELMIRA, N. Y.

